# Supplementary material for: Multi‐dataset identification of innovative feature genes and molecular mechanisms in keratoconus
Source: J Cell Mol Med. 2024 Sep 19;28(18):e70079. doi: 10.1111/jcmm.70079 (PMC11412914; doi:10.1111/jcmm.70079)
Supplement: Supplementary file 2 — Material S2. [file JCMM-28-e70079-s002.zip › 7.GSEA analysis/5.RNF217-AS1/KEGG_PORPHYRIN_AND_CHLOROPHYLL_METABOLISM.html]

Details for gene set KEGG\_PORPHYRIN\_AND\_CHLOROPHYLL\_METABOLISM[GSEA]

|  || Dataset | RNF217-AS1.group.cls#h\_versus\_l |
| Phenotype | group.cls#h\_versus\_l |
| Upregulated in class | h |
| GeneSet | KEGG\_PORPHYRIN\_AND\_CHLOROPHYLL\_METABOLISM |
| Enrichment Score (ES) | 0.27333006 |
| Normalized Enrichment Score (NES) | 0.8763853 |
| Nominal p-value | 0.65625 |
| FDR q-value | 1.0 |
| FWER p-Value | 1.0 |
Table: GSEA Results Summary

  

Fig 1: Enrichment plot: KEGG\_PORPHYRIN\_AND\_CHLOROPHYLL\_METABOLISM      
 Profile of the Running ES Score & Positions of GeneSet Members on the Rank Ordered List

  

| SYMBOL | TITLE | RANK IN GENE LIST | RANK METRIC SCORE | RUNNING ES | CORE ENRICHMENT || 1 | UGT1A8 | na | 901 | 0.455 | 0.0951 | Yes |
| 2 | ALAS2 | na | 2048 | 0.346 | 0.1541 | Yes |
| 3 | UGT2B4 | na | 3115 | 0.286 | 0.1995 | Yes |
| 4 | UGT1A6 | na | 4124 | 0.248 | 0.2365 | Yes |
| 5 | UGT2B11 | na | 5963 | 0.184 | 0.2326 | Yes |
| 6 | UGT2B28 | na | 7074 | 0.151 | 0.2408 | Yes |
| 7 | HMOX1 | na | 7285 | 0.145 | 0.2733 | Yes |
| 8 | UGT2B10 | na | 9434 | 0.097 | 0.2371 | No |
| 9 | UGT2A3 | na | 10034 | 0.086 | 0.2427 | No |
| 10 | UGT2A1 | na | 14187 | 0.028 | 0.1307 | No |
| 11 | HCCS | na | 14713 | 0.022 | 0.1214 | No |
| 12 | BLVRB | na | 15523 | 0.014 | 0.1018 | No |
| 13 | CPOX | na | 15977 | 0.009 | 0.0911 | No |
| 14 | FTH1 | na | 16991 | -0.001 | 0.0623 | No |
| 15 | BLVRA | na | 17691 | -0.008 | 0.0443 | No |
| 16 | FTMT | na | 18246 | -0.013 | 0.0318 | No |
| 17 | MMAB | na | 19383 | -0.024 | 0.0055 | No |
| 18 | GUSB | na | 19854 | -0.029 | -0.0005 | No |
| 19 | HMOX2 | na | 19911 | -0.029 | 0.0056 | No |
| 20 | UROD | na | 20523 | -0.035 | -0.0026 | No |
| 21 | PPOX | na | 20764 | -0.037 | 0.0005 | No |
| 22 | ALAS1 | na | 20839 | -0.038 | 0.0085 | No |
| 23 | EARS2 | na | 21410 | -0.044 | 0.0038 | No |
| 24 | ALAD | na | 23689 | -0.071 | -0.0430 | No |
| 25 | HMBS | na | 24032 | -0.076 | -0.0326 | No |
| 26 | UROS | na | 25759 | -0.103 | -0.0550 | No |
| 27 | UGT2B7 | na | 26756 | -0.121 | -0.0515 | No |
| 28 | FECH | na | 26882 | -0.123 | -0.0223 | No |
| 29 | UGT2B15 | na | 26916 | -0.124 | 0.0097 | No |
| 30 | COX15 | na | 29186 | -0.181 | -0.0074 | No |
| 31 | COX10 | na | 30828 | -0.235 | 0.0078 | No |
| 32 | CP | na | 33377 | -0.393 | 0.0389 | No |
Table: GSEA details [plain text format]

  

Fig 2: KEGG\_PORPHYRIN\_AND\_CHLOROPHYLL\_METABOLISM      
 Blue-Pink O' Gram in the Space of the Analyzed GeneSet

  

Fig 3: KEGG\_PORPHYRIN\_AND\_CHLOROPHYLL\_METABOLISM: Random ES distribution      
 Gene set null distribution of ES for **KEGG\_PORPHYRIN\_AND\_CHLOROPHYLL\_METABOLISM**
